# Retrospective Analysis With Propensity Score Matching of Peripheral T-Cell Lymphoma Treated Frontline With Brentuximab Vedotin and Chemotherapy

**DOI:** 10.1093/oncolo/oyad068

**Published:** 2023-03-27

**Authors:** John M Burke, Nicholas Liu, Kristina S Yu, Michelle A Fanale, Andy Surinach, Carlos Flores, Julie Lisano, Tycel Phillips

**Affiliations:** US Oncology Hematology Research Program, Rocky Mountain Cancer Centers, Aurora, CO, USA; Health Economics and Outcomes Research, Seagen Inc., Bothell, WA, USA; Health Economics and Outcomes Research, Seagen Inc., Bothell, WA, USA; Health Economics and Outcomes Research, Seagen Inc., Bothell, WA, USA; Real-World Evidence Analytics, Genesis Research, Hoboken, NJ, USA; Evidence Strategy, Genesis Research, Hoboken, NJ, USA; Health Economics and Outcomes Research, Seagen Inc., Bothell, WA, USA; Department of Internal Medicine, Division of Hematology and Oncology, University of Michigan Medical School, Ann Arbor, MI, USA

**Keywords:** brentuximab vedotin, peripheral T-cell lymphoma, real-world evidence, claims analysis, treatment patterns

## Abstract

**Background:**

Since Food and Drug Administration approval of brentuximab vedotin in combination with cyclophosphamide, doxorubicin, and prednisone (A + CHP) as initial therapy for previously untreated CD30-expressing peripheral T-cell lymphoma (PTCL), there has been limited research on real-world patient characteristics, treatment patterns, and clinical outcomes.

**Methods:**

We retrospectively analyzed claims of patients with PTCL treated with frontline A + CHP or CHOP (cyclophosphamide, doxorubicin, vincristine, prednisone) using the Symphony Health Solutions database. Adults with International Classification of Diseases-9/10 PTCL diagnosis codes who initiated A + CHP or CHOP between November 2018 and July 2021 were included. A 1:1 propensity score matching analysis was performed that adjusted for potential confounders between groups.

**Results:**

A total of 1344 patients were included (A + CHP, n = 749; CHOP, n = 595). Before matching, 61% were men; median age at index was 62 (A + CHP) and 69 (CHOP) years. The most common A + CHP-treated PTCL subtypes were systemic anaplastic large cell lymphoma (sALCL; 51%), PTCL-not otherwise specified (NOS; 30%), and angioimmunoblastic T-cell lymphoma (AITL; 12%); the most common CHOP-treated subtypes were PTCL-NOS (51%) and AITL (19%). After matching, similar proportions of patients treated with A + CHP and CHOP received granulocyte colony-stimulating factor (89% vs. 86%, P = .3). Fewer patients treated with A + CHP received subsequent therapy than CHOP overall (20% vs. 30%, *P* < .001) and specifically with the sALCL subtype (15% vs. 28%, *P* = .025).

**Conclusions:**

Characteristics and management of this real-world PTCL population who were older and had a higher comorbidity burden than that in the ECHELON-2 trial demonstrate the importance of retrospective studies when assessing the impact of new regimens on clinical practice.

## Teaser

This article reports the results of the first real-world study of patient and treatment characteristics since FDA approval of brentuximab vedotin in combination with CHP for previously untreated systemic anaplastic large cell lymphoma or other CD30-expressing peripheral T-cell lymphomas.

**Implications for Practice**

This administrative claims database analysis of patients with peripheral T-cell lymphoma (PTCL) treated with frontline brentuximab vedotin, cyclophosphamide, doxorubicin, and prednisone (A + CHP) or cyclophosphamide, doxorubicin, vincristine, and prednisone (CHOP) provides the first insights into patients’ characteristics, PTCL subtypes, granulocyte colony-stimulating factor use, and subsequent therapies since the November 2018 FDA approval of frontline A + CHP for CD30-expressing PTCLs. Differences in the characteristics and management of this real-world population with PTCL compared with a clinical trial population underscore the importance of real-world studies in assessing the impact of novel regimens on clinical practice and identifying areas for further education of practitioners.

## Introduction

Peripheral T-cell lymphomas (PTCLs) are a group of rare, aggressive non-Hodgkin lymphomas (NHLs) originating from post-thymic or mature T cells and natural killer (NK) cells.^1^ PTCLs account for ~10% of new NHL cases, equating to 8,000-12,000 new PTCL cases in the US in 2020.^2,3^ The World Health Organization has described >25 different types of PTCLs, and distinct molecular signatures are being investigated to identify candidates for targeted treatments.^3^ CD30 is universally expressed in systemic anaplastic large cell lymphoma (sALCL) and expressed in ~50% of non-sALCL subtypes.^4,5^

Brentuximab vedotin (BV) is a CD30-directed antibody-drug conjugate approved by the US Food and Drug Administration (FDA) in November 2018 for the treatment of adults with previously untreated sALCL or other CD30-expressing PTCLs, including angioimmunoblastic T-cell lymphoma (AITL) and PTCL not otherwise specified (PTCL-NOS), in combination with cyclophosphamide, doxorubicin, and prednisone (A + CHP).^6^ In the phase III ECHELON-2 study (NCT01777152), at a median follow-up of 47.6 months, frontline (1L) treatment with A + CHP versus CHOP improved median progression-free survival (PFS; 62.3 vs. 23.8 months), 5-year PFS (51.4% vs. 43.0%; HR, 0.70; 95% CI: 0.53-0.91), and 5-year overall survival (OS) (70.1% vs. 61.0%; HR, 0.72; 95% CI: 0.53-0.99) in 452 patients with CD30-expressing PTCL.^7^ Median OS was not reached with either treatment. Additionally, safety profiles were comparable for A + CHP and CHOP. Of note, the trial was weighted toward sALCL, with 316 patients (70%) having sALCL.

The standard 1L therapy for PTCL that does not express CD30 is cyclophosphamide, doxorubicin, vincristine, and prednisone (CHOP) or a CHOP‐like regimen, despite minimal evidence from high-quality prospective studies.^8^ CHOP and CHOP-like regimens result in complete remission (CR) rates of ~65%,^9,10^ 2-year PFS rates of 35%-47%, and 2-year OS of 53% for previously untreated PTCL.^11^ Although options exist for salvage therapy, responses tend to occur in a minority of patients, with reported 5-year OS of 37%.^12,13^

Although double-blind, randomized controlled trials (RCTs) are the gold standard for evaluating drug efficacy and safety, RCTs often lack external validity because they limit enrollment to patients who may be younger or have fewer comorbidities than patients in real-world settings.^14^ This is relevant with PTCL, where >50% of patients are aged ≥62 years at diagnosis, and among patients aged ≥70 years with PTCL, comorbidities are common, with 35%-40% of these patients having a Charlson Comorbidity Index (CCI) score of ≥2.^15,16^ In addition, as PTCLs are rare, with large subtype heterogeneity, subtype analyses in RCTs are challenging due to small sample sizes.

Real-world data can be used to overcome these limitations; however, baseline patient and disease characteristics may differ between identified cohorts. Propensity score matching (PSM) is an analytic technique used to balance baseline patient and disease characteristics (as done in an RCT), minimize confounding bias, and allow for between-group comparisons.^17,18^

To describe patient characteristics, PTCL subtypes, and treatment patterns in US patients with PTCL treated 1L with A + CHP or CHOP outside of a clinical trial, we conducted the first study of real-world patient and treatment characteristics since FDA approval of BV in November 2018 for previously untreated sALCL or other CD30-expressing PTCLs in combination with CHP (ie, A + CHP).

## Methods

### Data Source

This retrospective study used medical and pharmacy claims from the Symphony Health Solutions Patient-Level Claims database to identify patients with PTCL treated with 1L A + CHP or CHOP. Symphony Health Patient Level Claims data consist of claims submitted by physicians and pharmacies for reimbursement of services rendered to patients using CMS1450 and CMS1500 forms for medical claims and National Council for Prescription Drug Programs forms for pharmacy claims. The database captures a substantial portion of total medical activity in the US, is geographically representative, and includes all plans and payment types (cash, commercial, Medicare, Medicaid, and assistance programs). Patients are uniquely identified and can be tracked for >10 years across all settings. Over 95% of claims are linked to a unique practitioner.

### Study Design

**Figure 1.**
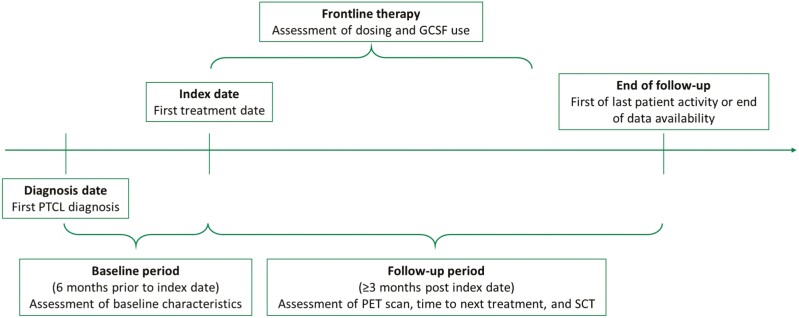
Study design. Abbreviations: GCSF, granulocyte colony-stimulating factor; PET, positron emission tomography; PTCL, peripheral T-cell lymphoma; SCT, stem cell transplantation.

The study observation period was from November 2013 to October 2021, during which time patients were required to have a PTCL diagnosis (Fig. 1). The patient identification period spanned from November 2018 to July 2021, during which cohorts were identified based on first antineoplastic treatment. Patients were assigned a diagnosis date (date of first PTCL claim) and an index date (date of first administration of any drug component for A + CHP or CHOP during the patient identification period).

The patient identification period began on November 1, 2018, based on the FDA approval date of A + CHP. Patient demographics were assessed over the 6-month baseline period prior to the index. July 31, 2021, was selected as the end of the patient identification period to allow at least 3-month follow-up post index. The follow-up period varied for all patients and spanned from index to last patient activity observed or end of study (October 31, 2021), whichever occurred earlier.

### Study Population

Eligible patients had a PTCL diagnosis (International Classification of Diseases [ICD], 9th Revision, Clinical Modification: 200.6x & 202.7x; ICD, 10th Revision, Clinical Modification: C84.4x to C84.7x, C84.Zx, C84.9x, C86.1, C86.2, C86.5, and C91.5x) during the study observation period, along with the following:

- 1 inpatient or 2 outpatient claims, ≥1 day apart, with a PTCL diagnosis
- ≥1 claim for antineoplastic treatment on or after the diagnosis date
- age ≥18 years as of index
- at least 6 months of activity before and at least 3 months of activity after index
- an index date during the identification period
- A + CHP or CHOP treatment within 21 days post index

Patients with multiple PTCL subtype diagnoses were excluded.

### Patient Characteristics

Demographic characteristics assessed at index were age, sex, and geographical region. PTCL subtype was reported based on the PTCL subtype at diagnosis. Subtypes included sALCL (anaplastic lymphoma kinase [ALK] positive [ALK+] and ALK negative [ALK−]), human T-lymphotropic virus (HTLV)-1-associated adult T-cell lymphoma/leukemia (ATLL), AITL, enteropathy-associated T-cell lymphoma, hepatosplenic T-cell lymphoma, other mature T/NK-cell lymphomas, and PTCL-NOS. Time metrics included patients’ activity time pre- and post-index. CCI scores were assessed during the 6-month baseline period based on the presence of an ICD-9/10 code for certain cardiovascular, pulmonary, gastrointestinal, renal, and hepatic conditions, as well as rheumatic disease, diabetes without/with chronic complications, dementia, hemiplegia or paraplegia, cerebrovascular disease, and HIV/AIDS (Table 1).

**Table 1.** Summary of demographic features and treatment characteristics unmatched and matched cohorts.

	Unmatched cohort				Matched cohort			
	Overall	A + CHP	CHOP	*P*-value	Overall	A + CHP	CHOP	*P*-value
	*N* = 1344	*n* = 749	*n* = 595		*N* = 1190	*n* = 595	*n* = 595	
Age at index, years, median (IQR)	66 (55, 74)	62 (49, 72)	69 (61, 76)	<.001	64 (53, 72)	58 (46, 67)	69 (61, 76)	<.001
Age at index, years (categorized), *n* (%)				<.001				<.001
18-39	123 (9.2)	100 (13.4)	23 (3.9)		123 (10.3)	100 (16.8)	23 (3.9)	
40-59	319 (23.7)	218 (29.1)	101 (17.0)		319 (26.8)	218 (36.6)	101 (17.0)	
60 or older	902 (67.1)	431 (57.5)	471 (79.2)		748 (62.9)	277 (46.6)	471 (79.2)	
Sex, *n* (%)				.008				<.001
Female	522 (38.8)	267 (35.6)	255 (42.9)		430 (36.1)	175 (29.4)	255 (42.9)	
Male	822 (61.2)	482 (64.4)	340 (57.1)		760 (63.9)	420 (70.6)	340 (57.1)	
US region, *n* (%)				.9				.7
Northeast	227 (16.9)	122 (16.3)	105 (17.6)		201 (16.9)	96 (16.1)	105 (17.6)	
Midwest	315 (23.4)	181 (24.2)	134 (22.5)		286 (24.0)	152 (25.5)	134 (22.5)	
South	569 (42.3)	316 (42.2)	253 (42.5)		502 (42.2)	249 (41.8)	253 (42.5)	
West	224 (16.7)	124 (16.6)	100 (16.8)		193 (16.2)	93 (15.6)	100 (16.8)	
Unknown	9 (0.7)	6 (0.8)	3 (0.5)		8 (0.7)	5 (0.8)	3 (0.5)	
PTCL subtype, *n* (%)								
sALCL (ALK status unspecified)	1 (<0.1)	0 (0)	1 (0.2)		1 (<0.1)	0 (0)	1 (0.2)	
sALCL ALK−	247 (18.4)	214 (28.6)	33 (5.5)		202 (17.0)	169 (28.4)	33 (5.5)	
sALCL ALK+	193 (14.4)	166 (22.2)	27 (4.5)		182 (15.3)	155 (26.1)	27 (4.5)	
PTCL-NOS	525 (39.1)	221 (30.0)	304 (51.1)		472 (39.7)	168 (28.2)	304 (51.1)	
AITL	201 (15.0)	91 (12.1)	110 (18.5)		174 (14.6)	64 (10.8)	110 (18.5)	
Adult T-cell lymphoma/leukemia (HTLV-1-associated)	62 (4.6)	25 (3.3)	37 (6.2)		52 (4.4)	15 (2.5)	37 (6.2)	
Enteropathy-associated T-cell lymphoma	21 (1.6)	4 (0.5)	17 (2.9)		20 (1.7)	3 (0.5)	17 (2.9)	
Hepatosplenic T-cell lymphoma	9 (0.7)	2 (0.3)	7 (1.2)		8 (0.7)	1 (0.2)	7 (1.2)	
Mature T/NK-cell lymphoma	62 (4.6)	16 (2.1)	46 (7.7)		59 (5.0)	13 (2.2)	46 (7.7)	
Other mature T/NK-cell lymphomas	23 (1.7)	10 (1.3)	13 (2.2)		20 (1.7)	7 (1.2)	13 (2.2)	
Baseline activity (months), median (IQR)	71 (62, 80)	72 (62, 80)	70 (62, 80)	.6	70 (62, 80)	70 (62, 79)	70 (62, 80)	.2
Follow-up activity (months), median (IQR)	13 (7, 22)	15 (7, 23)	12 (7, 21)	.014	14 (7, 23)	16 (8, 24)	12 (7, 21)	<.001
CCI score, median (IQR)	1.00 (0.00, 2.00)	1.00 (0.00, 2.00)	1.00 (0.00, 2.00)	.002	1.00 (0.00, 2.00)	0.00 (0.00, 1.00)	1.00 (0.00, 2.00)	<.001
No. of Charlson comorbidities, *n* (%)				.022				<.001
No comorbidities	620 (46.1)	372 (49.7)	248 (41.7)		567 (48)	319 (53.6)	248 (41.7)	
1 comorbidity	383 (28.5)	200 (26.7)	183 (30.8)		349 (29)	166 (27.9)	183 (30.8)	
2 comorbidities	166 (12.4)	91 (12.1)	75 (12.6)		140 (12)	65 (10.9)	75 (12.6)	
≥3 comorbidities	175 (13.0)	86 (11.5)	89 (15.0)		134 (11)	45 (7.6)	89 (15.0)	
Charlson comorbidity, *n* (%)								
Myocardial infarction	63 (4.7)	42 (5.6)	21 (3.5)	.1	50 (4.2)	29 (4.9)	21 (3.5)	.3
Congestive heart failure	125 (9.3)	64 (8.5)	61 (10.3)	.3	96 (8.1)	35 (5.9)	61 (10.3)	.008
Peripheral vascular disease	122 (9.1)	57 (7.6)	65 (10.9)	.045	95 (8.0)	30 (5.0)	65 (10.9)	<.001
Cerebrovascular disease	65 (4.8)	32 (4.3)	33 (5.5)	.3	54 (4.5)	21 (3.5)	33 (5.5)	.13
Dementia	10 (0.7)	1 (0.1)	9 (1.5)	.007	9 (0.8)	0 (0)	9 (1.5)	.004
Chronic pulmonary disease	250 (18.6)	130 (17.4)	120 (20.2)	.2	219 (18)	99 (16.6)	120 (20.2)	.13
Rheumatic disease	50 (3.7)	23 (3.1)	27 (4.5)	.2	42 (3.5)	15 (2.5)	27 (4.5)	.084
Peptic ulcer disease	35 (2.6)	19 (2.5)	16 (2.7)	>.9	32 (2.7)	16 (2.7)	16 (2.7)	>.9
Mild liver disease	140 (10.4)	73 (9.7)	67 (11.3)	.4	121 (10)	54 (9.1)	67 (11.3)	.2
Diabetes without chronic complications	259 (19.3)	134 (17.9)	125 (21.0)	.2	214 (18)	89 (15.0)	125 (21.0)	.008
Diabetes with chronic complications	87 (6.5)	48 (6.4)	39 (6.6)	>.9	60 (5.0)	21 (3.5)	39 (6.6)	0.024
Hemiplegia or paraplegia	11 (0.8)	7 (0.9)	4 (0.7)	.8	8 (0.7)	4 (0.7)	4 (0.7)	>.9
Renal disease	147 (10.9)	65 (8.7)	82 (13.8)	.004	114 (9.6)	32 (5.4)	82 (13.8)	<.001
Moderate or severe liver disease	8 (0.6)	1 (0.1)	7 (1.2)	.025	8 (0.7)	1 (0.2)	7 (1.2)	.069
AIDS/HIV	12 (0.9)	4 (0.5)	8 (1.3)	.2	11 (0.9)	3 (0.5)	8 (1.3)	.2
Neutropenia on or after 1L	608 (45.2)	335 (44.7)	273 (45.9)	.7	541 (45.5)	268 (45.0)	273 (45.9)	.8

Abbreviations: 1L, frontline; A + CHP, brentuximab vedotin plus cyclophosphamide, doxorubicin, and prednisone; AITL, angioimmunoblastic T-cell lymphoma; ALK, anaplastic lymphoma kinase; CCI, Charlson Comorbidity Index; CHOP, cyclophosphamide, doxorubicin, vincristine, and prednisone; IQR, interquartile range; NK, natural killer; PET, positron emission tomography; PTCL, peripheral T-cell lymphoma; PTCL-NOS, PTCL-not otherwise specified; sALCL, systemic anaplastic large cell lymphoma.

### Definition of Line of Therapy

Previously untreated patients with PTCL were selected for the study. To further determine treatment groups (A + CHP and CHOP) based on 1L therapy, all treatments that began within 21 days of index were considered 1L therapy. Patients were considered to receive a subsequent line of therapy (LOT) if any of the following occurred:

◦ Treatment discontinuation—a gap of >45 days with no treatment claims. The end of a LOT was defined as the last treatment claim before the gap in treatment.
◦ Treatment switch—a claim for a new treatment not included in the LOT occurring ≥22 days post-index. The end of a LOT was defined as the last claim before the new treatment.

### Outcomes

Dose was defined as the unique number of drug administration days for any component of A + CHP or CHOP as 1L treatment, separated by ≥1 day. If a patient had 2 days of consecutive doses, only 1 day was counted as a valid dose. A BV cycle was defined as every BV dose administered in the 1L setting based on a dosing schedule of day 1 of each 21-day cycle.

Primary use of granulocyte colony-stimulating factors (GCSFs) was defined as ≥1 GCSF claim on or within 5 days of index. GCSF use was evaluated only in the 1L setting. Patients with no evidence of primary GCSF use were eligible for secondary use. Secondary GCSF use was defined as the presence of ≥1 GCSF claim >5 days from index. Patients with no GCSF claims during 1L treatment were classified as having no prophylactic GCSF use.

Time to subsequent LOT was defined as the time between index and start of next LOT. Patients not progressing to a next LOT in their follow-up were censored at the end of their continuous activity or end of study, whichever occurred first. The second LOT was also examined, with number and percentage of patients with types of treatment provided.

Stem cell transplantation (SCT) was identified via the ICD-9/10 procedure, Current Procedural Terminology, Healthcare Common Procedure Coding System, and Diagnosis-Related Group codes during the follow-up period.

### Propensity Score Matching

A + CHP- and CHOP-treated patients, selected after inclusion criteria were applied, were matched using propensity scores to account for differences in baseline characteristics between cohorts. The propensity score, defined as the probability of being treated with A + CHP or CHOP conditional on the patient’s baseline characteristics, was obtained using a logistic regression model. Variables included in the model were patient demographics (age at index, sex, geographical region), baseline clinical characteristics (CCI score), and follow-up time (months).

A + CHP- and CHOP-treated patients were matched 1:1. PSM was performed using the greedy nearest neighbor technique.

### Statistical Analyses

Descriptive statistics were used to describe demographic, clinical, tumor subtype, and treatment characteristics for the unmatched and matched cohorts. Summary statistics were reported, including frequencies and percentages for categorical variables, means and standard deviations (SDs), and medians and interquartile ranges (IQRs) for continuous variables. *P*-values were provided for A + CHP and CHOP comparisons using Wilcoxon rank-sum test for continuous variables and chi-square test of independence or Fisher’s exact test for categorical variables.

Kaplan-Meier methods were used to calculate time to subsequent LOT, with median survival time and 95% CI examined. The log-rank *P*-value was reported for the statistical difference between A + CHP and CHOP cohorts. A Cox proportional hazards model was used to assess the impact of treatment groups on next LOT. In addition to A + CHP and CHOP as the cohort indicator, variables not balanced between cohorts after PSM were also controlled in the model. Hazard ratios (HRs), 95% CIs, and *P*-values were provided. Statistical analyses were performed using SAS version 9.3 (SAS Institute Inc., NC, USA).

## Results

### Patient Characteristics: Unmatched Cohort

**Figure 2.**
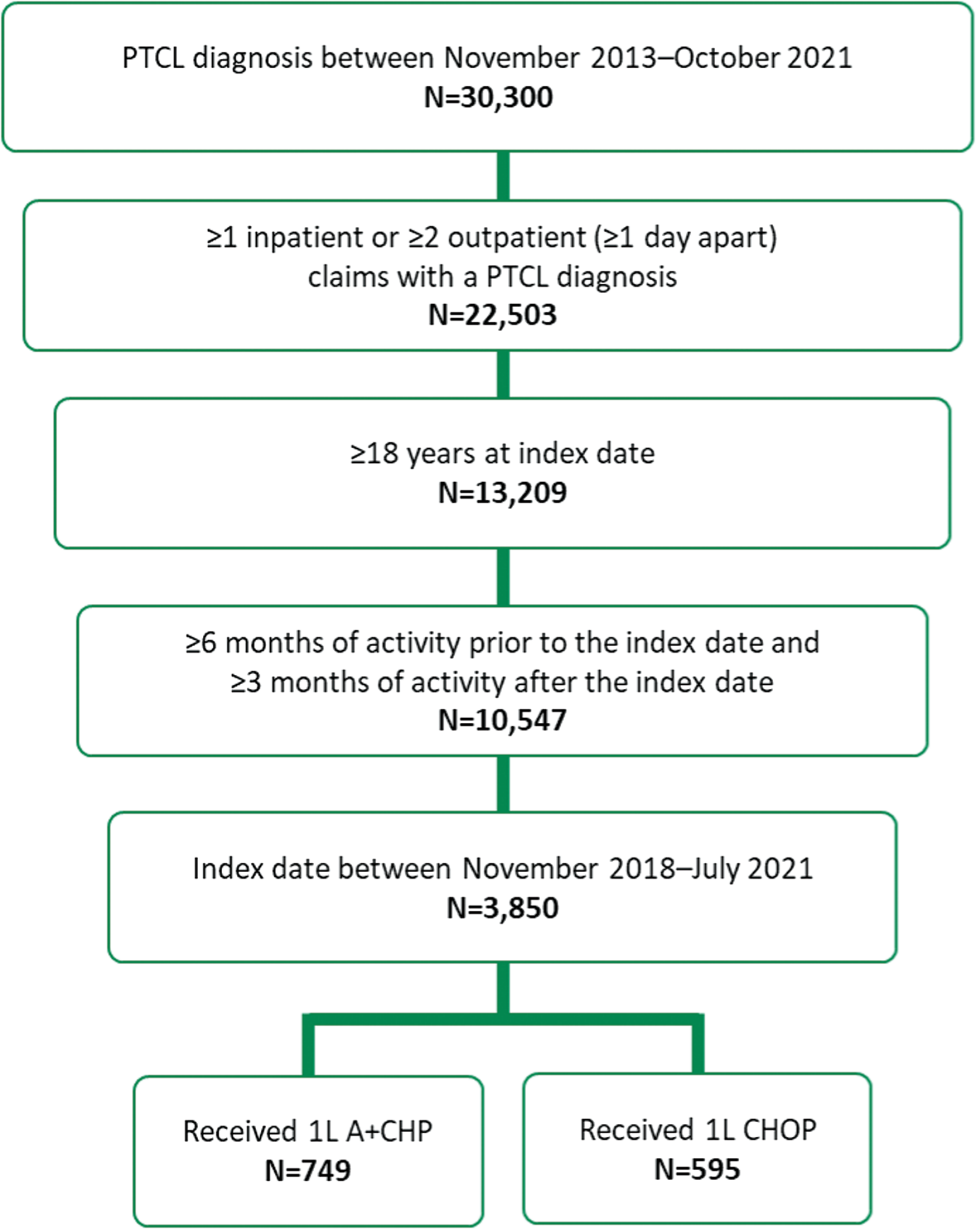
Summary of cohort selection. Abbreviations: 1L, frontline; A + CHP, brentuximab vedotin plus cyclophosphamide, doxorubicin, and prednisone; CHOP, cyclophosphamide, doxorubicin, vincristine, and prednisone; PTCL, peripheral T-cell lymphoma.

Of the 30,300 patients in the Symphony Health Solutions Patient-Level Claims database with a PTCL diagnosis between 2013 and 2021, 1344 patients met selection criteria and were included in the study (A + CHP, *n* = 749; CHOP, *n* = 595; Fig. 2).

The median patient age was 66 years at index, with patients treated with CHOP significantly older than those treated with A + CHP (69 vs. 62 years, *P* < .001) (Table 1). Overall, more men than women were included in the study (61% vs. 39%), and 42% of the patients resided in the South.

**Figure 3.**
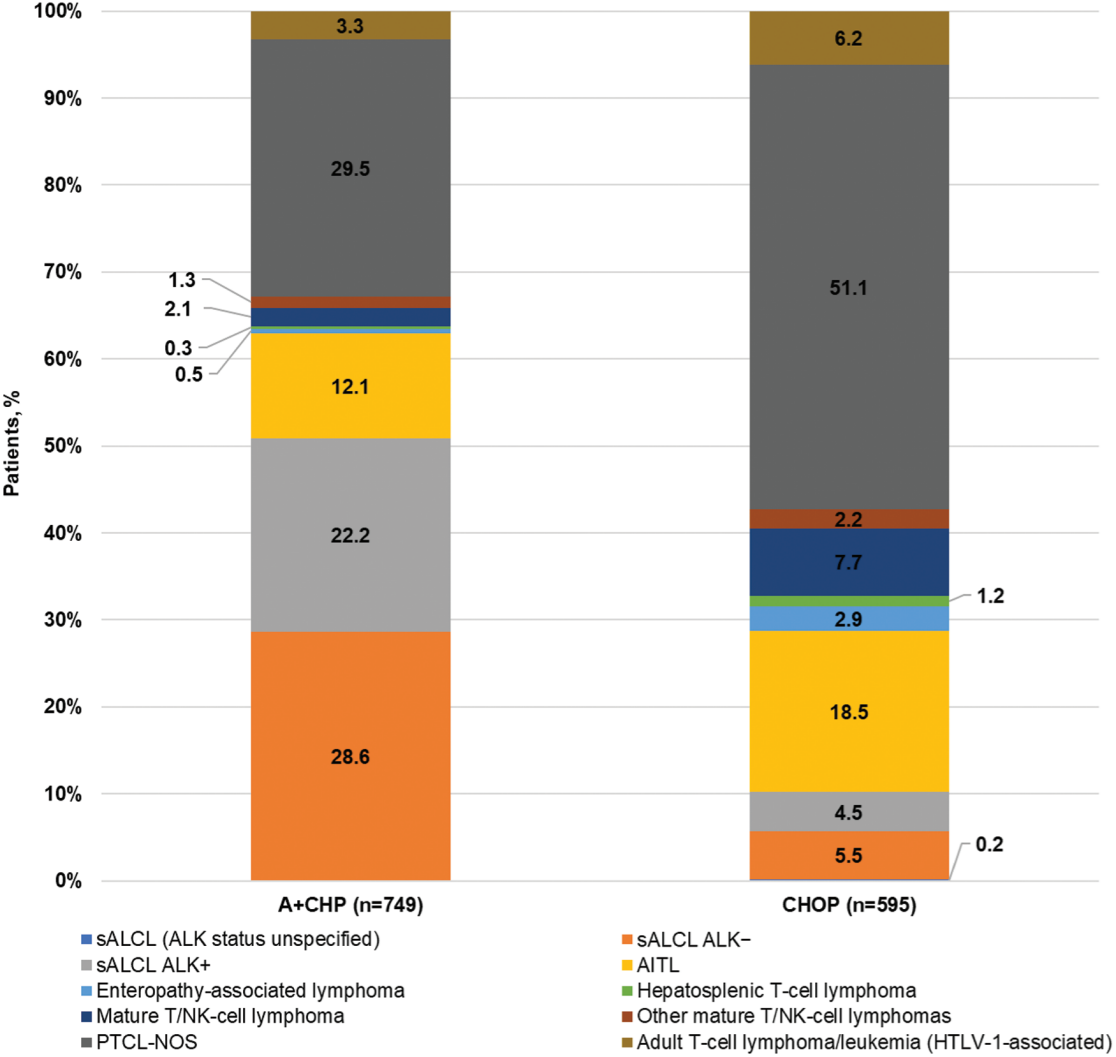
Distribution of PTCL subtypes among patients treated with frontline A + CHP or CHOP, unmatched cohort. Abbreviations: A + CHP, brentuximab vedotin plus cyclophosphamide, doxorubicin, and prednisone; AITL, angioimmunoblastic T-cell lymphoma; ALK−, anaplastic lymphoma kinase-negative; AKL+, anaplastic lymphoma kinase-positive; CHOP, cyclophosphamide, doxorubicin, vincristine, and prednisone; NK, natural killer; PTCL, peripheral T-cell lymphoma; PTCL-NOS, PTCL not otherwise specified; sALCL, systemic anaplastic large cell lymphoma.

The median follow-up time was longer for the A + CHP than the CHOP group (15 vs. 12 months; *P* = .014). The median CCI score was 1 for the overall population, with 54% of patients having ≥1 comorbidity during the baseline period; the proportion of patients with comorbidities was lower for A + CHP than CHOP (50% vs. 58%; *P* = .022). The most common comorbidities were diabetes without chronic complications (19%) and chronic pulmonary disease (19%). Differences in PTCL subtypes were found, with sALCL and PTCL-NOS most common in the A + CHP group and PTCL-NOS and AITL most common in the CHOP group (Fig. 3).

### Characteristics of A + CHP and CHOP Patients: Matched Cohort

Following PSM, all 595 patients treated with CHOP were matched to a patient treated with A + CHP. After matching, age, sex, CCI score, and follow-up time were found to be imbalanced between the 2 groups; matching did not significantly change the relative proportion of PTCL subtypes in the A + CHP and CHOP groups (Table 1). Patients treated with A + CHP versus CHOP were younger (*P* < .001) and had longer median follow-up times (16 vs. 12 months; *P* < .001).

### Treatment Dosing and GCSF Use: Matched Cohort

Patients treated with A + CHP received a mean (SD) of 4.6 (2.0) doses, and those treated with CHOP received 4.1 (1.9) doses in the 1L setting (*P* < .001). A total of 46% of patients treated with 1L A + CHP and 33% with 1L CHOP received ≥6 doses (*P* < .001).

In the A + CHP group, the median (IQR) number of BV cycles was 5 (3, 6), with 42% of patients receiving ≥6, 18% receiving 5, 11% receiving 4, and 29% receiving ≤3 cycles. GCSF use was similar between the A + CHP and CHOP groups (89% vs. 86%; *P* = .3), with 86% of patients receiving GCSF as primary prophylaxis. Pegfilgrastim was the most used GCSF in both groups (A + CHP, 94%; CHOP, 92%; *P* = .8). The median (IQR) duration of prophylactic GCSF use was similar for the A + CHP and CHOP groups (90 [44, 106] vs. 84 [42, 106] days; *P* = .4).

### Second-Line Therapies: Matched Cohorts

#### PTCL Population

**Figure 4.**
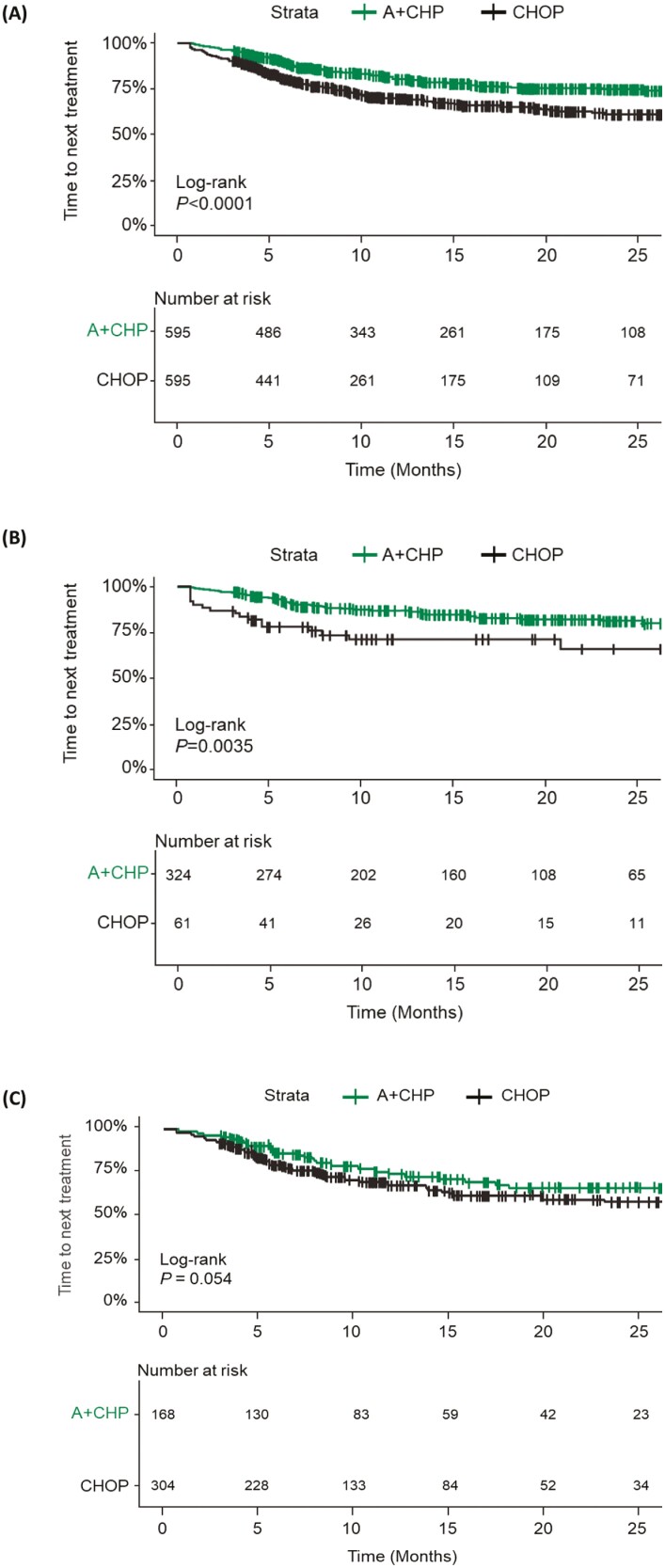
Time to next treatment among all patients with PTCL (**A**) and the subgroups with sALCL (**B**) and PTCL-NOS (**C**) treated with A + CHP and CHOP, Kaplan-Meier results (matched cohort). Abbreviations: A + CHP, brentuximab vedotin plus cyclophosphamide, doxorubicin, and prednisone; CHOP, cyclophosphamide, doxorubicin, vincristine, and prednisone; PTCL, peripheral T-cell lymphoma; sALCL, systemic anaplastic large cell lymphoma.

In total, 119 (20%) patients treated with A + CHP and 176 (30%; *P* < .001) treated with CHOP received a second LOT during the follow-up period, among whom 32% (38/119) of those treated with A + CHP and 24% (43/176) of those treated with CHOP received a BV-containing regimen. Patients treated with A + CHP versus CHOP were less likely to receive second-line therapy during the follow-up period (log-rank *P* < .0001; Fig. 4A).

**Figure 5.**
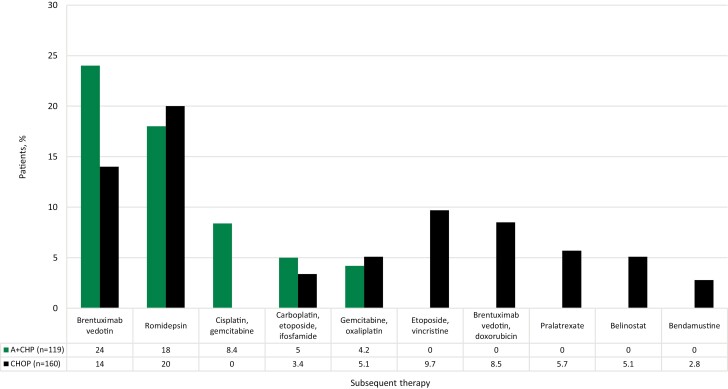
Second-line therapy for PTCL following A + CHP or CHOP (matched cohort). Abbreviations: A + CHP, brentuximab vedotin plus cyclophosphamide, doxorubicin, and prednisone; CHOP, cyclophosphamide, doxorubicin, vincristine, and prednisone; PTCL, peripheral T-cell lymphoma.

Results from the Cox proportional hazards model, after controlling for age, sex, follow-up time, and CCI score, were similar and showed that patients treated with CHOP versus A + CHP had a higher likelihood of receiving second-line therapy during the follow-up period (HR: 1.64; 95% CI, 1.26, 2.13; *P* < .001). The most administered second-line therapies were BV (24% A + CHP; 14% CHOP) and romidepsin (18% A + CHP; 20% CHOP) (Fig. 5).

#### sALCL Population

Subsequent LOTs were also examined for patients with sALCL. Among these patients, 15% (49/324) of those treated with A + CHP and 28% (17/61; *P* = .025) of those treated with CHOP received a second LOT during the follow-up period. Among those receiving second-line therapy, 37% (18/49) of patients treated with 1L A + CHP and 65% (11/17) treated with 1L CHOP received a BV-containing regimen.

Patients treated with A + CHP versus CHOP were less likely to receive a second LOT during the follow-up period (log-rank *P* = .0035; Fig. 4B). In the Cox proportional hazards model, after controlling for age, sex, follow-up time, and CCI score, patients treated with CHOP versus A + CHP had a higher likelihood of receiving second-line therapy during the follow-up period (HR: 1.92; 95% CI, 0.99, 3.72; *P* < .053).

BV was the most common second-line therapy (24% A + CHP; 41% CHOP). Cisplatin + gemcitabine (14%) and romidepsin (10%) were also used second-line following 1L A + CHP.

#### PTCL-NOS Population

Subsequent LOTs were also examined for patients with PTCL-NOS. Of patients with PTCL-NOS, 24% (41/168) of those treated with A + CHP and 32% (97/304; *P* = .86) of those treated with CHOP received a second LOT during the follow-up period. Among those receiving second-line therapy, 29% (12/41) of patients treated with 1L A + CHP and 22% (21/97) treated with 1L CHOP received a BV-containing regimen.

Patients treated with A + CHP versus CHOP were less likely to receive a second LOT during the follow-up period (log-rank *P* = .054; Fig. 4C). In the Cox proportional hazards model, after controlling for age, sex, follow-up time, and CCI score, patients treated with CHOP versus A + CHP had a higher likelihood of receiving second-line therapy during the follow-up period (HR: 1.43; 95% CI, 0.97, 2.11; *P* = .067).

BV and romidepsin were the most common second-line therapies (both 24% A + CHP; 13% and 23% CHOP). Etoposide (7.3%) and gemcitabine + oxaliplatin (7.3%) were also used second-line following 1L A + CHP.

### SCT: Matched Cohort

During the follow-up period, 100 (8.4%) patients received an SCT; by treatment, the proportion of A + CHP and CHOP patients undergoing SCT did not differ (9.9% vs. 6.9%; *P* = .076) (Table 2). The time from 1L treatment to SCT was ~6 months in both cohorts (*P* = .7). Most patients undergoing SCT received an autologous SCT.

**Table 2.** Stem cell transplantation following 1L treatment with A + CHP or CHOP for PTCL: matched cohort.

	Overall	A + CHP	CHOP	*P*-value
	*N* = 1190	*n* = 595	*n* = 595	
Received SCT, *n* (%)	100 (8.4)	59 (9.9)	41 (6.9)	.076
PTCL Subtype				.022
PTCL—NOS	37 (37.0)	16 (27.1)	21 (51.2)	
AITL	23 (23.0)	15 (25.4)	8 (19.5)	
sALCL ALK−	13 (13.0)	11 (18.6)	2 (4.9)	
sALCL ALK+	13 (13.0)	11 (18.6)	2 (4.9)	
ATLL	4 (4.0)	3 (5.1)	1 (2.4)	
Enteropathy-associated lymphoma	3 (3.0)	1 (1.7)	2 (4.9)	
Mature T/NK-cell lymphoma	3 (3.0)	1 (1.7)	2 (4.9)	
Other mature T/NK-cell lymphoma	3 (3.0)	1 (1.7)	2 (4.9)	
Hepatosplenic T-cell lymphoma	1 (1.0)	0 (0)	1 (2.4)	
Time from 1L to first SCT (months), median (IQR)	6.2 (5.3, 8.4)	6.1 (5.4, 8.2)	6.5 (5.0, 8.7)	0.7
Type of SCT, *n* (%)				>.9
Autologous	87 (87.0)	51 (86.4)	36 (87.8)	
Allogeneic	13 (13.0)	8 (13.6)	5 (12.2)	

Abbreviations: 1L, frontline; A + CHP, brentuximab vedotin plus cyclophosphamide, doxorubicin, and prednisone; AITL, angioimmunoblastic T-cell lymphoma; ALK-, anaplastic lymphoma kinase-negative; AKL+, anaplastic lymphoma kinase-positive; ATLL, adult T-cell leukemia/lymphoma CHOP, cyclophosphamide, doxorubicin, vincristine, and prednisone; IQR, interquartile range; NK, natural killer; PTCL, peripheral T-cell lymphoma; PTCL-NOS, PTCL not otherwise specified; sALCL, systemic anaplastic large cell lymphoma; SCT, stem cell transplant.

## Discussion

This claims analysis of patients with PTCL treated with 1L A + CHP or CHOP in real-world settings provides the first insights into patient characteristics, PTCL subtypes, GCSF use, and subsequent therapies since FDA approval of A + CHP in November 2018.

Prior to PSM, we found that patients treated with A + CHP versus CHOP were younger at 1L treatment initiation and were followed longer post-initiation of 1L treatment. Prior to matching, differences in PTCL subtypes were found between groups. Most patients treated with A + CHP had sALCL (ALK+ and ALK−, 51%), PTCL-NOS (30%), or AITL (12%); in contrast, most patients treated with CHOP had PTCL-NOS (51%), AITL (19%), sALCL (ALK + and ALK–, 10%), or mature T/NK-cell lymphoma (8%).

To account for differences in baseline characteristics between the A + CHP and CHOP groups, we matched the cohorts using a 1:1, greedy nearest neighbor without replacement PSM algorithm, which was obtained using logistic regression models. Following matching, an imbalance in age, sex, CCI score, and follow-up time was found between the groups. Thus, a Cox proportional hazards model was conducted. In the matched cohort, the mean number of doses received was significantly higher with A + CHP than CHOP (4.6 vs. 4.1; *P* < .001), and significantly more patients treated with A + CHP than CHOP received ≥6 doses of 1L therapy (*P* < .001). The proportion of patients who used GCSF and the duration of GCSF use were similar between the A + CHP and CHOP groups; most GCSF was used as primary prophylaxis. Of note, it is conceivable that primary prophylaxis with GCSF was initiated after day 5 of cycle 1 in some cases; therefore, these results may underestimate the rate of primary GCSF prophylaxis and overestimate the rate of secondary GCSF prophylaxis.

For the overall population and for patients with sALCL and PTCL-NOS subtypes, patients treated with A + CHP versus CHOP were less likely to receive subsequent therapy during the follow-up period, suggesting that additional treatments were not needed. This result was confirmed using a Cox proportional hazards model that controlled for differences in age, sex, follow-up time, and CCI scores between the A + CHP and CHOP groups. Although patients with PTCL-NOS treated with A + CHP versus CHOP were not significantly less likely to receive subsequent therapy, results trended in favor of A + CHP. Factors that may have contributed to the lower need for subsequent therapies following A + CHP include: (1) the greater proportion of patients with an sALCL subtype, which has a more favorable prognosis than other PTCL subtypes, among those treated with A + CHP than CHOP and (2) the higher efficacy of A + CHP, as demonstrated in ECHELON-2, than CHOP in PTCL, particularly in patients with sALCL.^7,19^ Among patients with PTCL, approximately 1 in 3 treated with A + CHP was later retreated with a BV-containing regimen, while 1 in 4 patients treated with CHOP received a subsequent BV-containing regimen in a later LOT; the higher percentage of BV use as salvage in the A + CHP group was likely due to the universal CD30 expression in these patients.

Results from this real-world study among patients with PTCL demonstrate that patients are being retreated with a BV-containing regimen. Among patients with an sALCL subtype who received a subsequent LOT, 38% of patients treated with A + CHP and 65% of patients treated with CHOP received a BV-containing regimen. In patients with sALCL, which universally expresses CD30, the target of BV, BV salvage therapy was less common in the A + CHP group presumably because some patients were thought to be BV refractory, whereas the CHOP group contained no patients who were BV refractory.

A numerically higher proportion of patients treated with A + CHP than CHOP received an SCT (9.9% vs. 6.9%). As patients treated with A + CHP versus CHOP were younger and had fewer comorbidities, this difference may suggest more patients treated with A + CHP were eligible for SCT. These figures point to the relative underutilization of SCT in real-world patients with PTCL. Time to SCT from initiation of 1L therapy was 6.1 and 6.5 months with A + CHP and CHOP.

Real-world characteristics may differ from those captured in clinical trials as treatments are based on patient characteristics and on clinician and patient preferences, rather than on trial selection criteria.^20^ Some differences between this real-world analysis and the ECHELON-2 study were found. Patients in this analysis were older (median age, 66 years vs. 58 years, respectively) and had fewer SCTs (A + CHP, 9.9% vs. 22%; CHOP, 6.9% vs. 17%).^7,21^ There was a high comorbidity burden in the current analysis, with over half of the patients in both the A + CHP and CHOP cohorts having ≥ 1 comorbidity, which reflects the older population in this analysis.

Finally, A + CHP was used in a broad range of PTCL subtypes in this study, including several not included in ECHELON-2, such as NK/T-cell lymphomas. In addition, there was a higher proportion of patients with the sALCL subtype in the A + CHP versus CHOP group, certainly due to the universal expression of CD30, the target of BV, in sALCL.

### Limitations

The limitations of this study include those inherent to any retrospective claims study. First, clinical information is based only on diagnosis and procedure codes and is limited by corresponding caveats. For example, this study used diagnosis codes to identify PTCL cases, which may be prone to coding errors.

Second, since claims data are designed and collected for billing purposes, a lack of clinical completeness and accuracy may exist. In our study, an algorithm was designed to identify lines of therapies based on dosing schedule of the drugs included. However, special usage could be anticipated; for example, patients could have switched to a new drug due to toxicity after 21 days of treatment but continued the same line.

Third, although the Symphony data represent a substantial proportion of all medical claims in the US, selection bias may be an issue embedded in the patient population, which could have led to biased results. Specifically, patients with sALCL have more favorable outcomes versus patients with non-sALCL subtypes.^19,22-26^ In this analysis, more patients treated with A + CHP than CHOP had sALCL and were therefore more apt to have a favorable outcome.

In addition to limitations related to claims data, the observational retrospective study design also caused restrictions in our study. We were unable to account for differences in demographic (age, sex, and follow-up time) and clinical characteristics (CCI score) between A + CHP and CHOP cohorts using a PSM algorithm. Instead of imposing stricter criteria to achieve more balanced cohorts, we decided to account for these differences in our Cox proportional hazards model. Still, unmeasured confounders (eg, disease stage, CD30 testing, disease progression, response outcomes, and death) inherent to claims data and the nature of the study may have introduced bias.

And last, although the data provide important information about real-world treatment use and patient characteristics, results may not be generalizable to all patients with PTCL or to all practice settings.

## Conclusions

Consistent with real-world studies, this real-world population expanded on that evaluated in the ECHELON-2 trial, with patients in this real-world analysis being older and having a greater comorbidity burden.^20,21,27^ The high comorbidity burden seen in this analysis is potentially a reflection of the older population. Additionally, in this study, A + CHP was more commonly used than CHOP in patients with sALCL, a finding not unexpected due to the universal CD30 expression in this PTCL subtype and the superior outcomes reported for A + CHP versus CHOP in ECHELON-2. For PTCL subtypes in which CD30 is more variably expressed, A + CHP and CHOP were used with similar frequencies. CHOP was commonly used for the ATLL subtype, and A + CHP was used for subtypes not included in ECHELON-2, such as NK/T-cell lymphomas. Results from this real-world analysis also show that GCSF was used as primary prophylaxis in a large majority of patients in both cohorts. Finally, following treatment with 1L A + CHP or CHOP, patients treated with 1L A + CHP were less likely to receive subsequent therapy. Subsequent therapy with a BV-containing regimen was more common in patients treated with 1L A + CHP than CHOP, likely due to these patients having CD30-expressing tumors. Differences in characteristics and management of this real-world population with PTCL compared with a clinical trial population underscore the importance of real-world studies in assessing the impact of new regimens on clinical practice and identifying areas for further education of practitioners.

## Data Availability

The data underlying this article will be shared on reasonable request to the corresponding author.

## Prior Congress Presentations

Some of these data were presented at the American Society of Hematology Annual Meeting 2020 (Virtual Event; December 5-8, 2020; Publication Number: 3418) and the Pan Pacific Lymphoma Conference (Big Island, HI; August 9-13, 2021).
